# Management of prostate abscess in the absence of guidelines

**DOI:** 10.1590/S1677-5538.IBJU.2016.0472

**Published:** 2017

**Authors:** Haitham Abdelmoteleb, Fatima Rashed, Amr Hawary

**Affiliations:** 1 Great Western Hospital, Swindon, United Kingdom

**Keywords:** Prostate, Disease Management, Guidelines as Topic

## Abstract

In contemporary practice, the number of patients presenting with prostatic abscess have significantly declined due to the widespread use of antibiotics. However, when faced with the pathology, prostatic abscess tends to pose a challenge to clinicians due to the difficulty of diagnosis and lack of guidelines for treatment. Treatment consists of an array of measures including parenteral broad-spectrum antibiotic administration and abscess drainage.

## INTRODUCTION

Prostatic abscesses are a rare clinical entity in the current practice due to the widespread use of antibiotics. Management usually imposes a challenge to urologists, that is due to the difficult diagnosis, as it may mimic other diseases of the lower urinary tract and the lack of guidelines for treatment (1). Prostate abscess (PA) usually develops in immunocompromised patients including diabetic and HIV patients as a consequence of acute bacterial prostatitis (2). The reason for the lack of guidelines as regards to PA is that most of the published data in the literature are case reports due to the declining incidence of the disease nowadays.

The incidence of PA in men with HIV has been reduced by the use of antiretroviral therapy (ART), which has decreased the incidence of opportunistic infections, and also by the use of long-term antibiotics in HIV men with bacterial or atypical urinary tract infections (UTIs) (3). In their series of 209 HIV patients, Leport et al. found that the incidence of PA in their patients was 5.3% (4). Both HIV and gonococcal urethritis are sexually transmitted disease. In HIV positive patients, PCR done for urethral swabs showed that the presence of urethral HIV DNA was significantly associated with gonococcal urethritis (5).

The extensive use of antibiotics for the treatment of diverse pathological infections and the decrease of the gonococcal urethritis associated with urethral stenosis, which previously favoured chronic genitourinary infections, have, without doubt, had a great impact on the declining incidence and mortality from prostatic abscesses. This shift was due to the early diagnosis and treatment of the pelvic and prostatic infections mainly acute bacterial prostatitis. It is estimated that the frequency of prostatic abscess can be as high as 0.5% of urologic diseases and that the mortality rate is between 1% and 16%. The most common bacteria related with prostatic abscesses is E. coli, with an occurrence of up to 70% in such cases (6).

### Aetiology and pathogenesis

Some authors suggest that PA is mostly a complication of bacterial prostatitis whether acute or chronic, most commonly seen in men in their fifth or sixth decade but can occur at any age (7). Before the advent of modern antibiotic therapy, 75% of prostatic abscesses were attributable to Neisseria gonorrhoea, and the mortality rate was between 6% and 30% (8). Currently, Enterobacteriaceae, especially E. coli, are the predominant pathogens in acute bacterial prostatitis. Less commonly found organisms are Klebsiella sp, Proteus mirabilis, Enterococcus faecalis and Pseudomonas aeruginosa (9). More recently, there has been a rise in the reported cases of methicillin-resistant Staphylococcus aureus (MRSA) as the causative agent of PA in literature. Risk factors for MRSA infection include: urinary catheter use, health care exposure, history of genitourinary surgery, presence of comorbidities and increasing age (10).

As with most urinary tract infections, prostatic abscess tends to develop from urinary reflux from the urethra toward the prostatic acini, favoured by the different phases of ejaculation and micturition (11). This means that prostatic abscesses are made up of small micro abscesses that coalesce in order to form larger ones which, eventually, on their natural course, could complicate spontaneous drainage through the urethra (8). Haematogenous dissemination has also been described from a septic focus from respiratory, digestive, urinary tracts or of soft tissue. In these cases, the most frequent micro-organisms are S. aureus, M. tuberculosis, Escherichia coli and Candida sp (12).

Predisposing factors for PA include an indwelling catheter, instrumentation of the lower urinary tract, bladder outlet obstruction, acute and chronic bacterial prostatitis, chronic renal failure, hemodialysis, biopsy of the prostate, diabetes mellitus, cirrhosis, and, more recently, acquired immunodeficiency syndrome (6). Urologists should have a high index of suspicion of PA in those groups of patients which are the high risk groups.

### Clinical Presentation

Prostatic abscess can cause a diagnostic dilemma because, in the early stages, prostatic abscess shares signs and symptoms of others diseases of the lower urinary tract. Symptoms and clinical findings of prostatic abscess are extremely variable. Initially the disease manifests as dysuria, urgency, and frequency in 96% of the cases, fever in 30% to 72%, perineal pain in 20% and urinary retention in 1/3 of the patients (6, 8).

Prostatic abscess should be suspected in high risk group patients presenting with fever and persistent lower urinary tract symptoms that do not respond to antibiotics. A prostatic abscess may progress to spontaneous fistulisation into the urinary bladder, prostatic urethra, rectum, or perineum. In some cases, it can lead to severe sepsis and death (13). One of the theories proposed for development of sepsis in PA is panton-valentine leukocidin (PVL) which is a toxin produced by Staphylococcus aureus that leads to persistence of infection and aids in the spread of infection (14).

## DIAGNOSIS

### Clinical

The most typical sign of prostatic abscess is a severely tender prostate with areas of fluctuation on digital rectal examination, although those findings diverge between 16% and 88% (15). Other focal symptoms include perineal pain, obstructive urinary symptoms and/or acute urinary retention (13). Systemic signs could be fever, leucocytosis and leucocyturia as well (15).

## RADIOLOGICAL

### Trans-rectal Ultrasound (TRUS)

The diagnostic method of choice, which also serves as a treatment and follow-up tool for patients with prostatic abscess, is transrectal ultrasonography of the prostate. The most common finding is the presence of one or more hypoechoic areas, which contain thick pus primarily in the transition zone and in the central zone of the prostate, and which are permeated by hyperechogenic areas and distortion of the anatomy of the gland. Transrectal sonography usually underestimates the real periprostatic extension of the abscess (8).

### Tomography (CT)

The role of CT examinations is highlighted in diagnosing PA in cases of extraprostatic collections, as CT can accurately detect the extent of spread of the abscess, particularly to the ischiorectal fossa and perineum (16).

### Magnetic Resonance Imaging (MRI)

The use of MRI in PA hasn't been standardised and only limited studies are available. The MRI characteristics of an abscess are a hypointense signal on T1and hyperintense on T2 (17).

## TREATMENT

### Medical

Initial management entails the use of broad spectrum parenteral antibiotics. This is usually feasible as a single treatment in cases of monofocal abscess cavity <1cm in diameter. An abscess that fails to respond quickly to antibiotics with no signs of clinical improvement needs surgical intervention and drainage of the abscess with or without urine diversion (18). Usually, two weeks are needed before antibiotic treatment are deemed a failure and further surgical intervention would be warranted (19).

### Surgical

Several methods have been proposed for surgical drainages all with reported efficacy and feasibility; these are ultrasound guided drainage, transurethral drainage or open drainage (20–22).

### Ultrasound guided aspiration

There is a preference for minimally invasive procedures such as TRUS-guided aspiration or transperineal ultrasound guided aspiration. These procedures are considered as the standard procedure for drainage of PA as they are easy to perform under local anaesthesia, have low morbidity and can be repeated in case of failure or incomplete drainage (23, 24). Culture of pus that is aspirated is important because pathogens isolated are often different from those found in urine culture (25).

The first of two percutaneous methods to drain PA is the transrectal approach that utilises a transrectal ultrasound (TRUS) to guide a needle through the rectal wall and into the PA for drainage. This procedure is performed under local anaesthesia with the patient lying in the left lateral decubitus position. Lavage following drainage allows for antibiotics to be introduced directly into the post-drainage cavity (26). TRUS is usually safe to perform except in a few cases where TRUS is contraindicated such as in patients with severe haemorrhoids, anal fistulas, fissures or after abdominoperineal resection (27).

Regarding the outcome of TRUS-guided aspiration, only few studies are available due to the rarity of the condition. In their series, Gogus et al. and Collada et al. had a similar success rate following TRUS-guided aspiration of 83.3%. Success was defined as complete resolution of PA on subsequent US and complete resolution of PA after second TRUS guided aspiration respectively. The reasons for failure were abscess size >3cm, anechoic appearance and ultrasonographically heterogeneous. Transurethral drainage was used following failure of TRUS-guided aspiration and was successful (23, 28).

The other approach is the transperineal route that also entails the use of TRUS to guide a needle puncturing the perineum into the prostatic abscess. This is usually done under general anaesthesia but local anaesthesia can be used. The patient is placed in the lithotomy position and a needle is advanced from the perineum into the prostate. Following complete drainage of the abscess, a guidewire is placed into the cavity and dilatation of the puncture tract is achieved via the Seldinger technique. A loop catheter is then placed for further drainage and is left in place for several days. Varkarakis et al. reported a high success rate with a complete resolution of PA after transperineal drainage (22).

### Transurethral

If the abscess recurs or cannot be completely evacuated, transurethral deroofing is a more appropriate approach, leading to better drainage of the abscess cavity with early recovery of the patient (29). The site of the abscess cavity can be pre-operatively anticipated with the findings from digital rectal examination, transrectal ultrasonography, and CT scans. Additionally, the release of pus to the prostatic urethra, by intra-operative prostatic massage, can indicate the site of the abscess. Another method is to induce pus release to the prostatic urethra by creating several incisions with a Colling's knife in the expected site of the abscess, thus avoiding excessive resection of prostatic tissues. Once the site of the abscess has been localised, proper deroofing of the cavity is performed by resection of prostatic tissues around the cavity's neck (21).

Previously being the standard approach by urologists to treat PA, transurethral drainage has been replaced by the less invasive percutaneous drainage (30). However, transurethral deroofing of PAs is still employed for persistent abscesses that recur despite minimally-invasive treatment (31).

### Open drainage

**Figure 1:**
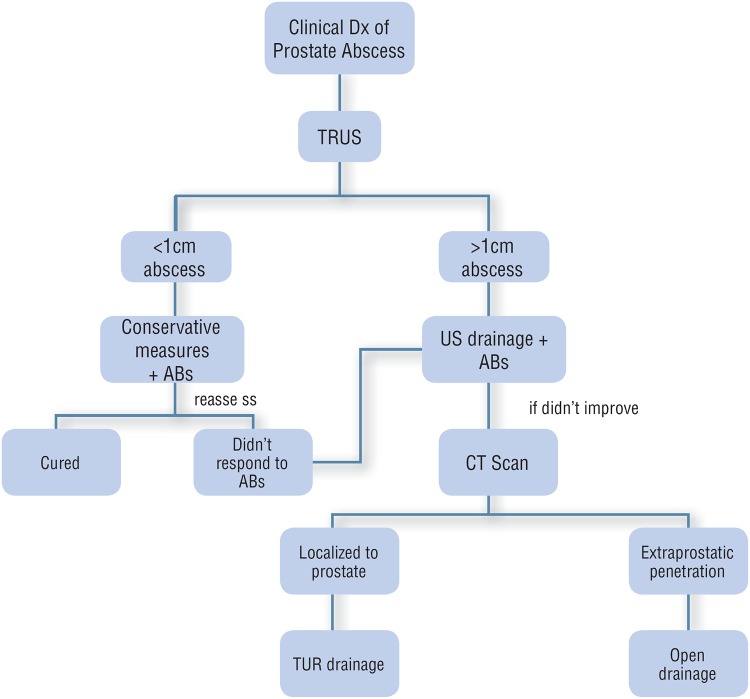
Algorithm for management of prostate abscess. Dx: Diagnoses, ABs: Antibiotics, US: Ultrasound, CT: Computed Tomography, TUR: Trans-urethral

Very rarely open surgical drainage might be required in patients with extraprostatic involvement (29). This is in the form of transperineal incision and drainage in cases where the abscess has penetrated through the levator ani muscle (13). Based on literature review we developed an algorithm for management of prostatic abscess (Figure-1).

EAU Guidelines (32) states that in the case of a prostatic abscess, both drainage and conservative treatment strategies appear feasible (29). When managing prostatic abscess, size does matter; in one study, conservative treatment was successful if the abscess cavities were < 1cm in diameter, while larger abscesses were better treated by single aspiration or continuous drainage (18).

## CONCLUSIONS

PA is a rare occurrence in current clinical practice due to the widespread use of antibiotics. It tends to affect individuals with impaired immune status. Adequate management leads to a better outcome. Due to lack of guidelines for management we recommend following local antibiotic policy as per microbiology guidance. Several methods are available for drainage that are tailored according to individual cases.
